# What do Cochrane systematic reviews say about interventions for treating psoriasis?

**DOI:** 10.1590/1516-3180.0271250618

**Published:** 2018-08-20

**Authors:** Rafael Leite Pacheco, Nicole Dittrich Hosni, Carolina de Oliveira Cruz Latorraca, Ana Luiza Cabrera Martimbianco, Daniela Vianna Pachito, Samira Yarak, Rachel Riera

**Affiliations:** I MD. Postgraduate Student, Evidence-Based Health Program, Universidade Federal de São Paulo (UNIFESP), and Assistant Researcher, Cochrane Brazil, São Paulo (SP), Brazil.; II Undergraduate Medical Student, Universidade Federal de São Paulo (UNIFESP), São Paulo, Brazil.; III MSc. Psychologist; Postgraduate Student, Evidence-Based Health Program, Universidade Federal de São Paulo (UNIFESP); and Assistant Researcher, Cochrane Brazil, São Paulo (SP), Brazil.; IV MSc, PhD. Physiotherapist; Postdoctoral Student, Evidence-Based Health Program, Universidade Federal de São Paulo (UNIFESP); and Volunteer Researcher, Cochrane Brazil, São Paulo (SP), Brazil.; V MD, MSc. Neurologist; Postgraduate Student, Evidence-Based Health Program, Universidade Federal de São Paulo (UNIFESP); and Assistant Researcher, Cochrane Brazil, São Paulo (SP), Brazil.; VI MD, MSc, PhD. Dermatologist and Adjunct Professor, Department of Dermatology, Escola Paulista de Medicina (EPM), Universidade Federal de São Paulo (UNIFESP), São Paulo (SP), Brazil.; VII MD, MSc, PhD. Rheumatologist; Adjunct Professor, Discipline of Evidence-Based Medicine, Escola Paulista de Medicina (EPM), Universidade Federal de São Paulo (UNIFESP); and Researcher, Cochrane Brazil, São Paulo (SP), Brazil.

**Keywords:** Review [publication type], Psoriasis, Evidence-based medicine, Evidence-based practice

## Abstract

**CONTEXT AND OBJECTIVE:**

Psoriasis is a common chronic inflammatory skin disease characterized by abnormal and increased growth of the cells that produce keratin and abnormal functioning of the immune system. We aimed to summarize the evidence available regarding interventions for patients with psoriasis.

**DESIGN AND SETTING:**

Review of systematic reviews, developed in the Discipline of Evidence-Based Medicine, Escola Paulista de Medicina, Universidade Federal de São Paulo.

**METHODS:**

A systematic search was conducted to identify Cochrane systematic reviews that fulfilled the eligibility criteria. Two authors screened titles and abstracts that had been retrieved through the search strategy. The results from all the Cochrane systematic reviews that were included were summarized and presented in a narrative synthesis.

**RESULTS:**

We included six Cochrane systematic reviews assessing interventions for treating psoriasis. The findings from high-quality evidence were that (a) etanercept reduced the psoriasis severity index, compared with placebo and (b) steroids plus vitamin D, compared with vitamin D alone, improved the skin clearance rate, as assessed by investigators, but was associated with a higher proportion of participants who dropped out due to adverse events. For all other comparisons, the quality of the evidence ranged from moderate to very low.

**CONCLUSION:**

This review included six Cochrane systematic reviews that provided evidence ranging in quality from unknown to high, regarding management of psoriasis. Further randomized controlled trials are imperative to reduce the uncertainties relating to several treatments that are already used in clinical practice.

## INTRODUCTION

Psoriasis is a common chronic inflammatory skin condition affecting about 1% to 2% of the general population in the United States and United Kingdom.^1^^,^^2^ The disease is characterized by abnormal and increased growth of the cells that produce keratin and abnormal functioning of the immune system, especially T lymphocytes.^3^

It may be triggered by several factors such as stress, alcohol consumption, drugs, smoking, sunlight, infections, local trauma, endocrine factors and genetic changes.^4^ Psoriasis can be identified in different clinical presentations or subtypes, including nail psoriasis, palmoplantar psoriasis, psoriatic arthritis, plaque psoriasis, psoriatic erythroderma and generalized pustular psoriasis.^5^ Around 20% of patients are diagnosed with severe types of the disease.^6^

There is no difference in prevalence between the sexes, although it is more common in adults than in children.^1^ It may be presented in association with a variety of comorbidities such as arthritis, depression and cardiovascular diseases.^7^ It can lead to fear of other reactions and social isolation, thus affecting the patients’ quality of life.^8^

There continues to be no cure for psoriasis.^9^ There are different options for treatments that can be used alone or in combination, such as systemic drugs, topical drugs and phototherapy.^10^ In decision-making, physicians need to consider the disease severity, the patients’ circumstances and the best evidence available.^11^

The aim of this review was to identify and summarize the evidence from Cochrane systematic reviews (SRs) relating to interventions for treating psoriasis.

## OBJECTIVE

To synthesize the evidence from Cochrane SRs regarding interventions for treating psoriasis.

## METHODS

### Design and setting

This study was a review of Cochrane SRs and was conducted within the Discipline of Evidence-based Medicine of Escola Paulista de Medicina, Universidade Federal de São Paulo (EPM-UNIFESP). This article was written for the section Cochrane Highlights. This initiative is a formal collaboration between the São Paulo Medical Journal and Cochrane, and it is supported by Cochrane Brazil. The aim of this initiative is to disseminate the evidence from Cochrane SRs.

### Inclusion criteria

#### Types of study

We only included SRs published in the Cochrane Database of Systematic Reviews. We excluded all protocols for SRs and withdrawn SRs. We also did not take previous versions of a single SR into consideration, or SRs that were being updated. We did not apply any date limit as an inclusion criterion.

#### Types of participants

The participants included were patients (regardless of age) who had been diagnosed with any form of psoriasis. SRs considering participants presenting psoriatic arthritis with no skin involvement were not included.

#### Types of intervention

We considered SRs assessing any intervention (whether pharmacological or not), which could either be single interventions or be in combination with other interventions, compared with any other intervention, an inactive comparator or no intervention (no treatment).

#### Types of outcomes 

We considered any outcome (clinical or laboratory), as evaluated in the SRs that were included.

### Search for reviews 

We conducted a broad search in the Cochrane Database of Systematic Reviews (via Wiley) on June 17, 2018. The full search strategy is presented in Table 1.

**Table 1:** Search strategy

#1 MeSH descriptor: [Psoriasis] explode all trees
#2 (Psoriasis) OR (Pustular Psoriasis of Palms and Soles) OR (Psoriases)
#3 #1 OR #2
#4 #3 Filter: in Cochrane Reviews

### Selection of reviews 

Two authors (RLP and DVP) screened the titles and abstracts independently. Any disagreements were resolved by consulting a third author (ALCM). The SRs that met the inclusion criteria were selected and summarized by three authors (COCL, RR, RLP).

### Presentation of results

We used a qualitative synthesis (narrative approach) to present the results from the search and from the SRs that were included.

## RESULTS

### Search results

Our search strategy retrieved 124 references. After screening the titles and abstracts, six systematic reviews were selected for full-text assessment.^12^^,^^13^^,^^14^^,^^15^^,^^16^^,^^17^ During this selection phase, two SRs were excluded because they were very outdated versions of two reviews that are currently being updated.^18^^,^^19^ Thus, we included six SRs for qualitative synthesis, given that they fulfilled our eligibility criteria.

### Reviews included

We present a summary of each SR that was considered. Details regarding the characteristics of the interventions, comparisons, outcomes and the quality of evidence are presented in Table 2.

**Table 2:** Characteristics of interventions, comparisons, outcomes and quality of evidence

Intervention	Comparators	Population	Main findings	GRADE^20^
Anti-tumor necrosis factor (etanercept) (0.8 to 50 mg per kilogram of body weight)^12^	Placebo	Children with moderate to severe plaque psoriasis who did not respond to, had a contraindication against, or did not tolerate other systemic therapies or photo chemotherapy	*Favored etanercept:* • Reduction of 50%, 75%, or 90% in the PASI index • Health-related quality of life	High Moderate
Pharmacological and non-pharmacological interventions, including infliximab, golimumab and superficial radiotherapy^13^	Placebo and active interventions	Participants with nail psoriasis	• Infliximab and golimumab were superior to placebo for improvement in nail score over the short and medium terms • Superficial radiotherapy (SRT) was superior to placebo in short-term treatment	NA NA
Narrow-band ultraviolet B phototherapy (NB-UVB)^14^	Oral PUVA, bath PUVA, topical PUVA, selective broad band ultraviolet B	Patients with chronic plaque psoriasis, palmoplantar psoriasis or guttate psoriasis	NB-UVB versus oral PUVA: No difference in PASI 75 or in discontinuation due to side-effects NB-UVB versus bath PUVA: The clearance rate favored bath PUVA NB-UVB versus topical PUVA: No difference in the clearance rate NB-UVB versus selective broad-band UVB: No difference in the clearance rate or in discontinuation due to side effects.	Low Low Low Low Low
Oral fumaric acid esters^15^	Placebo or methotrexate	All subtypes of psoriasis	*Favored fumaric acid esters, compared with placebo:* • PASI 75 *No difference between fumaric acid esters and methotrexate:* • PASI 75 • Adverse events	Very low Very low
Topical interventions including steroids and vitamin D^16^	Other topical interventions	Chronic plaque psoriasis	Multiple comparisons and analyses. Heterogeneity between studies prevented pooled analysis	NA
Topical interventions, including steroids and vitamin D^17^	Other topical interventions	Scalp psoriasis	Steroids versus vitamin D *Favored steroids* • Proportion of patients achieving “clearance”, according to investigators’ global assessment *Favored vitamin D* • Proportion of patients discontinuing due to adverse events Steroids plus vitamin D versus steroids alone *Favored steroids plus vitamin D* • Proportion of patients achieving “clearance”, according to investigators’ global assessment *No difference between groups* • Proportion of patients discontinuing due to adverse events Steroids plus vitamin D versus vitamin D *Favored steroids plus vitamin D* • Proportion of patients achieving “clearance”, according to investigators’ global assessment • Proportion of participants discontinuing due to adverse events.	Moderate Moderate Moderate Moderate High High

NA = not assessed; PASI = Psoriasis Area and Severity Index; PASI 75 = 75% reduction in the PASI index; NB-UVB = narrow-band ultraviolet B; PUVA = psoralen ultraviolet A photochemotherapy. *GRADE (Grading of Recommendations Assessment, Development and Evaluation) aims to assess the quality of the evidence. Outcomes are classified as providing the following: (a) high quality of evidence (high confidence that the estimated effect is near the true effect); (b) moderate quality of evidence (it is very likely that the estimated effect is close to the real effect but there is a possibility that it is not); (c) low quality of evidence (limited confidence in the effect estimate); or (d) very low quality of evidence (the true effect is likely to be substantially different from the estimated effect).

#### Anti-tumor necrosis factor agents for treating pediatric psoriasis

This review^12^ had the aim of evaluating the use of anti-tumor necrosis factor (TNF) agents in pediatric patients with psoriasis. One RCT (211 participants) was included. This trial assessed etanercept (at dosages ranging from 0.8 to 50 mg per kilogram of body weight), compared with placebo. One of the main outcomes consisted of investigator-assessed improvement, measured as the proportion of participants achieving 75% reduction in the Psoriasis and Severity Index (PASI). After 12 weeks, the number of patients achieving this reduction was higher in the etanercept group (60/106 participants) than in the placebo group (12/105). This represented a relative risk [RR] of 4.95; 95% confidence interval [95% CI] 2.83 to 8.65; one RCT; 211 participants; high quality of evidence. There was also an improvement favoring the etanercept group regarding the quality-of-life assessment using the Children’s Dermatology Life Quality Index (mean difference [MD] of 2.30; 95% CI 0.85 to 3.75; one RCT; 211 participants; moderate quality of evidence).

Regarding safety outcomes, three serious adverse events in the etanercept group were reported. The authors of the review concluded that use of etanercept seemed to be effective and safe for treating pediatric psoriasis. However, in relation to this conclusion, it needs to be borne in mind that the evidence came from a single industry-sponsored RCT. Further RCTs are imperative for reaching solid conclusions. For further details, refer to the original abstract, available from: http://cochranelibrary-wiley.com/doi/10.1002/14651858.CD010017.pub2/full.

#### Interventions for treating nail psoriasis

This review^13^ evaluated the efficacy and safety of the treatments for nail psoriasis and included 18 RCTs with 1,266 participants. It was not possible to pool the results because of the heterogeneity of many of the studies. The findings regarding the main outcome of improvement in nail score are presented below:

- Infliximab (5 mg/kg) was superior to placebo after medium-term treatment (57.2% improvement in nail score versus -4.1%; P < 0.001) and short-term treatment;
- Golimumab (50 mg and 100 mg) was superior to placebo after medium-term treatment (33% improvement in nail score versus 0% and 54% versus 0%, respectively; P < 0.001) and short-term treatment.
- Superficial radiotherapy (SRT) was superior to placebo after short-term treatment (20% improvement in nail score versus 0%; P = 0.03).
- Ciclosporin was similar to etretinate.
- Methotrexate was similar to ciclosporin.
- Ustekinumab was similar to placebo.
- 5-fluorouracil (1%) in Belanyx lotion as a vehicle was similar to Belanyx lotion alone.
- Tazarotene (0.1% cream) was similar to clobetasol propionate.
- Calcipotriol (50 µg/g) was similar to betamethasone dipropionate with salicylic acid.
- Calcipotriol (0.005%) was similar to betamethasone dipropionate.

Not all the studies included reported adverse events; those that did only reported mild adverse effects. Only one study reported the effect on quality of life, which limits the confidence in the results. 

For further details, refer to the original abstract, available from http://cochranelibrary-wiley.com/doi/10.1002/14651858.CD007633.pub2/full.

#### Narrow-band ultraviolet B phototherapy versus broad-band ultraviolet B or psoralen-ultraviolet A photochemotherapy for treating psoriasis

This SR^14^ had the aim of assessing the effects of different ultraviolet therapy interventions in patients with psoriasis. Thirteen RCTs (662 participants) were included. The quality of the evidence was assessed regarding four comparisons of narrow-band ultraviolet B (NB-UVB): against oral psoralen ultraviolet A photochemotherapy (PUVA), bath PUVA, topical PUVA and selective broad-band ultraviolet B. There was also a combined-therapy comparison: NB-UVB plus retinoid compared with PUVA plus retinoid.

In the comparison between NB-UVB and oral PUVA for treating chronic plaque psoriasis, there was no statistical difference between the groups regarding the proportions of the participants reaching 75% reduction in PASI (RR 0.91; 95% CI 0.63 to 1.32; one RCT; 51 participants; low quality of evidence). There was also no difference regarding the outcome of dropping out due to side effects (RR 0.71; 95% CI 0.20 to 2.54; three RCTs; 247 participants; low quality of evidence).

In the comparison between NB-UVB and bath PUVA for patients with chronic plaque psoriasis, the only outcome reported was the clearance rate (clearance was defined as no lesions of psoriasis or minimal residual activity). The clearance rate in studies that made comparisons between patients was significantly better in the bath PUVA group (RR 0.18; 95% CI 0.05 to 0.71; one RCT; 36 participants; low quality of evidence).

In the comparison between NB-UVB and topical PUVA for treating palmoplantar psoriasis, there was no statistical difference in the clearance rate (RR 0.09; 95% 0.01 to 1.56; one RCT; 50 participants; low quality of evidence).

In the comparison between NB-UVB and selective broad-band ultraviolet B for treating chronic plaque psoriasis, there was also no difference in the clearance rate (RR 1.40; 95% CI 0.92 to 2.13; one RCT; 100 participants; low quality of evidence). Nor was there any difference regarding dropping out due to side effects (RR 3.0; 95% CI 0.32 to 27.87; one RCT; 100 participants; low quality of evidence).

In the comparison of combined therapy of NB-UVB plus retinoid versus PUVA plus retinoid for patients with chronic plaque guttate psoriasis, there was also no difference in the clearance rate (RR 0.93; 95% CI 0.79 to 1.10; two RCTs; 90 participants; low quality of evidence). Nor was there any difference regarding the proportion of patients reaching 75% reduction in PASI (RR 0.89; 95% 0.59 to 1.35; 60 participants; one RCT; low quality of evidence).

The authors of this review concluded that the current evidence was heterogenous and needed to be interpreted with caution. All the evidence presented was from head-to-head comparisons and no inactive comparator was considered. The overall quality of the evidence was low because of imprecision and risk of bias. Further studies are imperative in order to confirm the efficacy and safety of these interventions for psoriasis.

For further details, refer to the original abstract, available from http://cochranelibrary-wiley.com/doi/10.1002/14651858.CD009481.pub2/full.

#### Oral fumaric acid esters for treating psoriasis

This review^15^ assessed the use of oral fumaric acid esters (FAE) for patients with psoriasis. Six RCTs (544 participants) were included and use of FAE was compared with use of placebo or methotrexate. The studies included presented high clinical and methodological diversity, and this prevented pooled analysis.

The PASI scale was used by the investigators in each RCT to assess improvement in different ways, and the authors of the SR decided to report the results from each RCT narratively. Five RCTs reported data from use of FAE versus placebo. In three of them (418 patients), the PASI score measurements showed that there were benefits from using FAE. The proportion of the patients who achieved 75% reduction in PASI was reported by two RCTs in which use of FAE was also favored. A single RCT (175 participants), published as an abstract, assessed quality of life using the Skindex-29 scale, but the numerical data was poorly reported and no formal analysis comparing the two arms of the study could be performed. The quality of the evidence for all these outcomes was considered low, following GRADE assessment.

Regarding the comparison of FAE versus methotrexate, only one RCT (51 participants) was included. There was no statistical difference between the proportions of patients who achieved 75% reduction in PASI, in this comparison (5/26 versus 6/26; RR 0.80; 95% CI 0.26 to 2.29; one RCT; 51 participants; very low quality of evidence). There was also no statistical difference in total PASI scores between the groups (MD 3.80; 95% CI 0.66 to 6.92; one RCT; 51 participants; very low quality of evidence).

Adverse events were poorly reported in general. From the RCT comparing FAE with methotrexate, it was reported that one patient in the FAE group discontinued the treatment due to adverse events while five patients in the methotrexate group dropped out, but this difference did not reach statistical significance (RR 0.89; 95% CI 0.77 to 1.03; one RCT; 54 participants; very low quality of evidence).

This SR made it clear that further studies are imperative for decreasing the uncertainties regarding the efficacy and safety of FAE for management of psoriasis. Further RCTs should be better planned and fully reported, to avoid further waste of research resources. The numerical data from all the RCTs included were reported in the full version of the SR. For further details, refer to the original abstract, available from http://cochranelibrary-wiley.com/doi/10.1002/14651858.CD010497.pub2/full.

#### Topical treatments for treating chronic plaque psoriasis

This SR^16^ assessed any topical intervention for chronic plaque psoriasis and included 177 RCTs (34,808 participants). In this summary, we present only the comparisons that the authors of the SR considered most clinically relevant.

For topical treatment, vitamin D analogues were significantly better than placebo in an investigator’s assessment of overall global improvement (IGA). Because many schemes for vitamin D administration were included, the authors of the SR presented results separated per scheme. They did not pool the results. For twice-daily use of becocalcidiol versus placebo, the standard mean difference [SMD] was -0.67 (95% CI -1.04 to -0.30; one RCT; 119 participants), which represents 0.8 points of improvement on an IGA scale from 0 to 6. For once-daily use of paricalcitol versus placebo, the SMD was -1.66 (95% CI -2.66 to -0.67; one RCT; 11 participants), which represents 1.9 points of improvement on an IGA scale from 0 to 6.

The authors of this SR also performed many analyses on corticosteroids alone or in combination with vitamin D compounds. In general, most corticosteroid interventions performed better then placebo, but this statement is not accurate for all corticosteroids. Each analysis needs to be considered when assessing the relevance of this intervention for treating chronic plaque psoriasis.

Many head-to-head comparisons were also included, and these data may be assessed in the full version of the SR. This SR did not include any evaluation of the quality of the evidence using the GRADE recommendations.

For further details and access to all the analyses, refer to the original abstract, available from http://cochranelibrary-wiley.com/doi/10.1002/14651858.CD005028.pub3/full.

#### Topical treatments for treating scalp psoriasis

This SR^17^ had the aim of evaluating all topical interventions for scalp psoriasis. Because of this wide eligibility criterion, 59 RCTs (11,561 participants) were included. In total, 15 comparisons were assessed. In this summary, we only report the following three comparisons, which the authors of this SR considered to be the “main comparisons”: steroids versus vitamin D; steroids plus vitamin D versus steroids; and steroids plus vitamin D versus vitamin D.

1. Steroids versus vitamin D:
  - Proportion of patients achieving “clearance”, according to the investigators’ global assessment: higher in the steroid group (392/1,392 versus 129/813; RR 1.82; 95% CI 1.52 to 2.18; 4 RCTs; 2,180 participants; moderate quality evidence).
  - Proportion of patients discontinuing due to adverse events: higher in the steroid group (15/1422 versus 44/869; RR 0.22; 95% CI 0.11 to 0.42; 4 RCTs; 2,291 participants; moderate quality of evidence).
2. Steroids plus vitamin D versus steroids:
  - Proportion of patients achieving “clearance”, according to the investigators’ global assessment: higher for combined therapy group (429/1,231 versus 357/1,243 participants; RR 1.22; 95% CI 1.08 to 1.36; four RCTs; 2,474 participants; moderate quality of evidence).
  - Proportion of patients discontinuing due to adverse events: no difference between groups (13/1211 versus 15/1,222 participants), representing a RR of 0.88 (95% CI 0.42 to 1.88; three RCTs; 2,433 participants; moderate quality of evidence).
3. Steroids plus vitamin D versus vitamin D:
  - Proportion of patients achieving “clearance”, according to the investigators’ global assessment: higher for combined therapy (442/1330 versus 96/678 participants; RR 2.28; 95% CI 1.87 to 2.78; four RCTs; 2,008 participants; high quality of evidence).
  - Proportion of participants discontinuing due to adverse events: higher for vitamin D alone (37/659 participants versus 14/1,311 participants; RR 0.19; 95% CI 0.11 to 0.36; three RCTs; 1,970 participants; high quality of evidence).

The authors of this SR concluded that use of steroids alone and combined with vitamin D presented more effective and safer results that did any other comparison assessed in the SR. These results should be considered in the context that all the results presented came from head-to-head comparisons and that no inactive treatment was considered in any of the three main comparisons. To check all other comparisons and analyses, refer to the full text. For further details, refer to the original abstract, available from http://cochranelibrary-wiley.com/doi/10.1002/14651858.CD009687.pub2/full.

## DISCUSSION

This review included six Cochrane systematic reviews that evaluated interventions for patients with psoriasis. Three of them had broad criteria for interventions and considered any intervention for a specific condition (scalp,^17^ chronic plaque^16^ and nail psoriasis^13^). The other three assessed specific interventions (oral fumaric acid esters,^15^ anti-TNF therapy^12^ and narrow-band ultraviolet B phototherapy^14^). Only one SR limited the population criterion, through only including pediatric patients.^12^

The fact that some SRs used broad criteria for interventions resulted in inclusion of a high number of randomized clinical trials (RCTs). Therefore, there was high diversity of populations, interventions and outcomes, and multiple analysis had to be performed. This should be taken into consideration, in making recommendations for practice.

**Figure 1:**
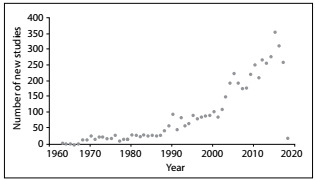
Number of studies indexed with “Psoriasis”[Mesh], published per year since 1963.

The number of systematic reviews published over the years has also been increasing. A rapid search of the literature, using the term “Psoriasis” [Mesh] and a filter for systematic reviews retrieved 5,310 abstracts from MEDLINE. The number of studies published per year since 1963 is presented in Figure 1.

This high number of published papers is a rough estimate. However, this volume of data does not necessarily mean that the quality of the data on interventions for treating psoriasis is good. The fact that Cochrane SRs follow rigid methodology and a special editorial process increases our confidence in the results included in this review.

Our search in the Cochrane library retrieved six protocols that in the future may present results of relevance for the clinical question of this review. These protocols, which have been published in the Cochrane Library, will assess the following: interventions for guttate psoriasis;^18^ methotrexate for psoriasis;^21^ indoor salt water baths followed by artificial ultraviolet B light for chronic plaque psoriasis;^22^ lifestyle changes for treating psoriasis;^23^ antistreptococcal interventions for guttate and chronic plaque psoriasis^19^; and complementary therapies for chronic plaque psoriasis.^24^

Regarding clinical implications, only two Cochrane SRs provided high-quality evidence relating to use of anti-TNF agents for psoriasis in pediatric patients and use of combined therapy of steroids and vitamin D for scalp psoriasis. More specifically, there was high-quality evidence showing that use of etanercept was related to higher numbers of participants reaching PASI 75 (75% reduction in the PASI index) than was use of placebo. There was also high-quality evidence showing that combined therapy of steroids plus vitamin D was better for the clearance rate than was vitamin D alone, as determined through the investigators’ global assessment, but that this combined therapy led to a higher proportion of participants dropping out due to adverse events. The evidence presented in Table 2 may provide a guide for clinical practice, but all healthcare decision-makers and patients need to be aware that future studies could produce drastically changed results, in relation to the current studies.

Regarding research implications, further studies are needed in order to reduce the uncertainties surrounding the effects from several interventions for treating psoriasis. The reporting on all RCTs needs to follow the recommendations of the Consolidated Standards for Reporting Trials (CONSORT).^25^ All studies need to be well designed and well conducted, in order to reduce imprecision and the risk of bias.

## CONCLUSION

This review included six Cochrane systematic reviews that provided quality of evidence for management of psoriasis that ranged from unknown to high. High quality of evidence was found favoring use of anti-TNF (etanercept) treatment for pediatric psoriasis and use of combined therapy of steroids plus vitamin D rather than vitamin D alone. Further randomized controlled trials are imperative for reducing the uncertainties relating to several treatments.
